# Evolutionarily new sequences expressed in tumors

**DOI:** 10.1186/1750-9378-1-8

**Published:** 2006-12-25

**Authors:** Andrei P Kozlov, Yuri P Galachyants, Ilya V Dukhovlinov, Nickolai A Samusik, Ancha V Baranova, Dmitry E Polev, Larisa L Krukovskaya

**Affiliations:** 1 The Biomedical Center, 8 Vyborgskaya St., St.Petersburg, 194044, Russia; 2 Center for the Study of Genomics in Liver Disearses, Molecular and Microbiology Department, George Mason University, Fairfax, USA

## Abstract

**Background:**

Earlier we suggested the concept of the positive evolutionary role of tumors. According to this concept, tumors provide conditions for the expression of evolutionarily new and/or sleeping genes in their cells. Thus, tumors are considered as evolutionary proving ground or reservoir of expression. To support this concept we have previously characterized *in silico *and experimentally a new class of human tumor-related transcribed sequences.

**Results:**

In this article we describe results of further studies of previously described tumor-related sequences. The results of molecular phylogeny studies, Southern hybridization experiments and computational comparison with genomes of other species are presented.

**Conclusion:**

These results suggest that these previously described tumor-related human transcripts are also relatively evolutionarily new.

## Background

In previous studies [1,2], we formulated the concept of the positive evolutionary role of tumors. According to this concept, tumors provide conditions for the expression of evolutionarily new and/or sleeping genes in their cells. Thus, tumors are considered as an evolutionary proving ground of expression.

In earlier work using the computational differential display approach, we identified a considerable number of human tumor-related expressed sequence tag (EST) clusters many of which had not been described previously [3]. Experimental data confirmed the results obtained *in silico*, i.e., the tumor-specificity of expression of these sequences [4].

To experimentally examine our prediction [1,2] that at least some tumor-related sequences are evolutionarily new, we performed Southern hybridization of our newly described tumor-related sequences with genomic DNA from different animal species. Hybridization was found only with human and orangutan DNA, with one exception in which a signal was also developed with chicken DNA.

We performed a search for ortholog sequences in fugu, tetraodon, zebrafish, frog, chicken, rat, mouse, cow, dog, macaque, and chimpanzee genomes using cross-species chained alignments. This search confirmed that our newly described tumor-related transcripts are relatively evolutionarily new, with some of their orthologs having originated in mammals and others in primates.

PCR experiments with specific primers were performed on a panel of DNAs from different primates. Amplified fragments were cloned and sequenced, and their molecular phylogeny was studied. The results show that these sequences form well-defined phylogenetic clusters which correspond to the phylogeny of primates as previously understood.

Taken together, our Southern hybridization, molecular phylogeny, and comparative genomics data support our prediction [1,2] that evolutionarily new and/or sleeping sequences may be specifically expressed in tumor cells.

## Results

### Transcribed sequences analyzed

Because of the constant rebuilding of the UniGene clusters and EST shuffling between them, we cannot follow the history of each cluster. Clusters very often do not mark a specific transcript, but a set of transcripts whose genome mapping regions are often neighboring but may be not overlapping. Therefore, we selected ESTs which were used for primer design in our previous investigations [3,4] and followed the history of their sequences in UniGene.

In this paper, we present analyses of the following ESTs (UniGene buid 185): [GenBank:AA166653], now in cluster Hs.426704 (former Hs. 154173); [GenBank:AL040372], now in cluster Hs.133294; [GenBank:AI952931] from cluster Hs.128594 (former Hs.67624); and [GenBank:AI792557] from cluster Hs.133107.

### PCR analysis and Southern hybridization

We performed Southern hybridization of [α-^32^P]-labeled sequence-specific fragments with genomic DNA from eleven different animal species: lamprey, fish, frog, chicken, pigeon, mouse, rat, guinea pig, sheep, horse, and human. Southern hybridization analysis reveals only homology sequences in chicken genome for AA166653-specific probe. In addition, we were able to demonstrate by Southern hybridization that a sequence homologous to the human AA166653-specific 11.2-kb fragment is present in orangutan DNA [see Additional file 1].

Therefore, we performed PCR amplification of sequence-specific fragments on the panel of primate DNAs. The results of PCR experiments and comparative genomics data obtained by homology analysis of these tumor-related sequences within primate DNA are presented in Table 1. As follows from the results shown in Table 1, sequences homologous to tumor-related human EST are found in a variety of primates.

**Table 1:** Results of PCR experiments* and comparative genomics analysis within primates.

Superfamily	Species/Transcript (EST Clusters)	#1	#2	#3	#4
*Platyrrhini*	*Lemur catta*	**+ **	**-**	**-**	**-**
New World monkeys	*Ateles fusciceps*	**+ **	**-**	**+ **	**+ **
	*Callimico goeldii*	**+ **	**-**	**+ **	**+ **
*Cercopitecoidea*	*Colobus guereza*	**+ **	**-**	**-**	**-**
Old World monkeys	*Erythrocebus patas*	**+ **	**-**	**-**	**+ **
	*Cercopithecus aethiops*	**+ **	**-**	**-**	**+ **
	*Macaca mulatta*	(+)**	**(+)**	**(+)**	**(+)**
*Hominoidea*	*Hylobates concolor*	**+ **	**-**	**+ **	**+ **
Apes and Human	*Pongo pygmaeus *(sumatran)	**+ **	**+ **	**+ **	**+ **
	*Pongo pygmaeus *(bornean)	**-**	**-**	**+ **	**+ **
	*Gorilla gorilla *(sample 1)	**+ **	**+ **	**+ **	**+ **
	*Gorilla gorilla *(sample 2)	**-**	**-**	+/-***	**+ **
	*Pan troglodytes *(sample 1)	**+ **	**+ **	**+ **	**(+)**
	*Pan troglodytes *(sample 2)	**+ **	**+ **	**+ **	**(+)**
	*Homo sapiens*	**+ **	**+ **	**+ **	**+ **

* DNA samples were ranged according to the existing classification of primates [5].

** Data in brackets are results of comparative genomics analysis.

*** Weak signal.

#1-AL040372, Hs.133294; #2-AA166653, Hs.426704; #3-AI952931, Hs.128594; #4-AI792557, Hs.133107

AL040372- and AI792557-specific tumor-related human sequences are found in the majority of primate species studied, even in the most archaic. The AI792557-homologous sequence is not found in lemurs and colobus monkeys. The AA166653-homologous sequence is present only in apes and macaques. The AI952931-homologous sequence is found in apes, new world monkeys, and macaques. No sequences discussed in this article could be amplified by PCR on a DNA evolutionary panel with genomic DNA from species non-primate species using the selected primers.

### Comparative genomics and bioinformatics analyses

Hs.133107 cluster consists of spliced mRNAs, but the studied sequence AI792557 is a short, unspliced EST and is mapped to an intronic region of Hs.133107. Analysis of cross-species chained alignments revealed that sequences homologous to AI792557 can be found in the genomes of the cow, dog, rat, mouse, rhesus, and chimpanzee with similarities of 60% to 72% in non-primate genomes and of 93% in rhesus and 98% in chimpanzee genomes (Table 2). Chimpanzee chromosome 8 contains a 400-bp region with a near-perfect homology to human EST AI792557, almost completely overlapping the 344-bp sequence of interest [see Additional file 2]. We also found an explanation for the reported absence of PCR signal in the chimpanzee genome: the AI792557-specific forward primer is disrupted by TTATC deletion located at the border of the segment of human-chimpanzee homology. It is of interest that both human and chimpanzee AI792557-like loci are 5'-flanked by an imperfect poly(t) repeat. Genomic sequences upstream of the poly(t) repeat and downstream of the 3' end of human-chimpanzee homologous segments do not possess any resemblance. Similarly, sequences corresponding to AI792557 were found in the genome of *Macaca mulatta*, via BLAT and chained alignments, but not in PCR experiments. Sequences that belong to the Hs. 128594 cluster represent human mRNA CACNA2D3 encoding for the voltage-dependent calcium channel protein alpha 2/delta 3 subunit. At the same time, our target 415-bp sequence, AI952931, is located in an intron of the CACNA2D3 gene. This EST has two exons and is transcribed from the strand opposite to the gene, as follows from direction of its splice sites consensus. The genomic sequence corresponding to the 315-bp 3'-exon can be found in genomes of cow, dog, rat, and mouse with a similarity of 64%–75%, and with almost perfect identity (93% and 99%) in the macaque and chimpanzee genomes ([see Additional file 2], Table 2). At the same time, sequences homologous to the 200-bp 3'-terminal fragment of this EST are found in the genomes of opossum (72% similarity) and chicken (61% similarity, Table 2). Only 14% and 16% of the human genomic sequence can be aligned with chicken and opossum orthologs, respectively (Table 2). Interestingly, the 120-bp sequence representing the 5'-exon sequence of AI952931 is entirely absent in all known genomes except human and macaque.

**Table 2:** Summary of cross-species homology analysis results

Sequence/Cluster	Human Position	Compared Genomes where Homology was found*	Aligned Bases between Genomes	% of Aligned Bases**	Matched Bases between Genomes	% of Matched Bases***	Human Sequence		Compared Sequence	
							
							Full length of Aligned Sequence	Unmatched Bases	Full length of Aligned Sequence	Unmatched Bases
AL040372/Hs.133294	chr1:153 308	opossum	965	**88.8**	505	**52.3**	1087	582	2068	1563
	314-153	mouse	753	**69.3**	537	**71.3**		550	771	234
	309 400	rat	1011	**93.0**	621	**61.4**		466	895	274
		cow	964	**88.7**	701	**72.7**		386	1916	1215
		dog	1060	**97.5**	714	**67.4**		373	1229	515
		rhesus	1086	**99.9**	1018	**93.7**		69	1073	55
		chimpanzee	1086	**99.9**	1071	**98.6**		16	1077	6
AI792557/HS.133107	chr8:129 160	mouse	401	**80.7**	242	**60.3**	497	255	576	334
	366-129	rat	412	**82.9**	249	**60.4**		248	561	312
	160 862	cow	418	**84.1**	279	**66.7**		218	405	126
		dog	414	**83.3**	297	**71.7**		200	624	327
		rhesus	496	**99.8**	460	**92.7**		37	488	28
		chimpanzee	496	**99.8**	486	**98.0**		11	490	4
AA166653/Hs.426704	chr2:132 864 310-	rhesus	516	**86.0**	454	**88.0**	600	146	712	258
	132 864 909	chimpanzee	599	**99.8**	594	**99.2**		6	603	9
AI952931/HS. 128594	chr3:54 641	chicken	227	**14.0**	138	**60.8**	1624	1486	555	417
	157-54	opossum	256	**15.8**	185	**72.3**		1439	262	77
	642 780	mouse	446	**27.5**	308	**69.1**		1316	439	131
		rat	658	**40.5**	420	**63.8**		1204	624	204
		cow	1330	**81.9**	980	**73.7**		644	1415	435
		dog	1302	**80.2**	977	**75.0**		647	1318	341
		rhesus	1623	**99.9**	1516	**93.4**		108	1635	119
		chimpanzee	1431	**88.1**	1413	**98.7**		211	1432	19

* Fugu, tetraodon, zebrafish, frog, chicken, rat, mouse, cow, dog, macaque, and chimpanzee genomes were analyzed.

** Percent of aligned bases were estimated as the ratio of aligned bases between genomes and the full length of the aligned human sequence.

*** Percent of matched bases were estimated as the ratio of matched and aligned bases between genomes.

A 450-bp tumor-related sequence corresponding to the non-coding cluster Hs.426704 (former Hs. 154173, core EST AA166653) is mapped to a human ribosomal DNA complete repeating unit. For this cluster, we used PCR primers specific to sequences located on chromosome 2. According to our experimental data [4], this sequence is expressed in carcinomas only. This sequence has not been found in any sequenced mammalian genome except rhesus and chimpanzee, with similarity levels of 88% and 99%, respectively ([see Additional file 2], Table 2). In the chimpanzee genome, the Hs.426704 locus underwent expansion, as it has been found in two locations on chromosome 13 as well as on chromosomes 18 and Y (Table 3).

**Table 3:** Duplications of tumour-related sequences studied in primate genomes

Mapping/Transcript (EST Cluster)	#1	#2	#3	#4
Original transcript mapping on chromosome in human genome	1	2	3	8
Human duplications and their mapping	1 (13)*	5 (12, 16, Y × 3)	0	0
The number of homologs in *P. troglodytes *genome and their mapping	2 (1, 14)	4 (13 × 2, 18, Y)	1 (2)	1 (7)
The number of homologs in *M. mulatta *genome**	2	6	1	1

* Chromosomes with sequence duplications are in brackets

** Mapping not shown as *M. mulatta *genome is available in draft version only.

#1-AL040372, Hs.133294; #2-AA166653, Hs.426704; #3-AI952931, Hs.128594; #4-AI792557, Hs.133107

Cluster Hs. 133294 corresponds to mRNA IQGAP3, which encodes a member of the Rho GTPase family of regulators involved in cytokinesis. Specifically, cluster Hs. 133294 includes an alternatively spliced isoform of the IQGAP3 gene that arises by retention of its 672-nt intron. Earlier [4], we demonstrated that this isoform is characterized by broad tumor-related and embryonal expression, thus representing a new carcinoembryonic transcript.

The AL040372-specific sequence corresponding to the tumor-related transcript of interest is mapped to the 3'-terminal intron and the 3'-UTR of IQGAP3 mRNA. Sequences with strong homology to this genomic region are present in macaque (94%) and chimpanzee (99%) genomes. Moreover, sequences with a similarity of 52%–73% to this genomic region have been found in opossum, mouse, rat, dog, and cow genomes (Table 2). Interestingly, the part of the 3'-UTR exonic sequence which is overexpressed in human tumors according to UniGene data is not present (or is highly divergent) in the mouse genome [see Additional file 2].

Summary data of the cross-species homology analysis of ESTs are presented in Table 2. Similar results were obtained when experimentally studied PCR fragments were analysed [see Additional file 2].

Using BLAT, we found that AL040372- and AA166653-homologous sequences have duplicates in the human and nonhuman primate genomes (Table 3).

### Molecular phylogenetic analysis

**Figure 1:**
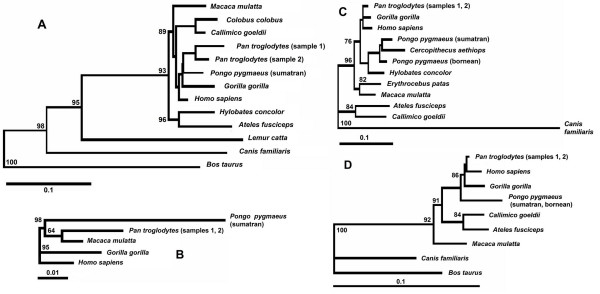
**Phylogeny trees of tumour-related sequences in primates**. Trees were constructed with the neighbor-joining method using pairwise deletion and tested with 1,000 bootstrap replicates, **(a) **Phylogeny of the AL040372-homologous sequence, which demonstrates a divergence of 8% ± 1.4% between *Homo sapiens *and *Lemur catta*.** (b) **Phylogeny analysis of the AA166653-homologous sequence. The maximum divergence in this cluster between *Homo sapiens *and *Pongo pygmaeus *is 7.8% ± 1.1%, and the divergence between *Homo sapiens *and *Pan troglodytes *is 4% ± 0.3%. **(c) **Phylogeny of the AI792557-homologous sequence among primates. The maximum divergence between *Homo sapiens *and *Ateles fusciceps *is 14.6% ± 1.3% for this sequence, **(d) **Phylogeny of the AI952931-homologous sequence among primates. The sequence divergence ranges from 0.9% to 7.8%. It was found that *Homo sapiens *and *Pan troglodytes *have 1.2% ± 0.4% divergence; the divergence between *Homo sapiens *and *Callimico *is 7.8% ± 1.2%.

Fig. 1a represents a phylogeny of AL040372-homologous sequences. The scale bar indicates the relative amount of change along branches. All-against-all BLAT searches among non-primate species sequences were conducted. Sequences with more than 70% identity were found in cow and dog genomes. These sequences were included in the phylogeny reconstruction. All primates except lemurs produce a well-supported monophyletic group which organizes a separate cluster on the phylogenetic tree. Among these fourteen sequences, lemur, dog, and cow form separate nodes. Fig. 1b shows a phylogenetic tree of AA166653-homologous sequences. These sequences were found in humans but in only four other primates. BLAT searches against non-primate genomes did not reveal any homologies. *Pongo *sequences form a distinct node. The phylogeny of AI792557-homologous sequences among primates is described in Fig. 1c. There are two separate clusters on the phylogenetic tree, one of which consists of new world monkeys (*Ateles *and *Callimico*), and the other of which includes apes and old world monkeys. *Erythrocebus *and macaques, which belong to the old world monkeys, form a separate branch. This phylogenetic tree corresponds well with the existing classification of primates. Fig. 1d represents the phylogeny of AI952931-homologous sequences. Sequences with near 80% similarity were found in the dog and cow genomes using BLAT. These sequences were included in the phylogeny reconstruction. On this tree, primate sequences formed a separate cluster that splits from the dog and cow node. Other algorithms (ML and MP) provide similar results in trees topology.

**Figure 2:**
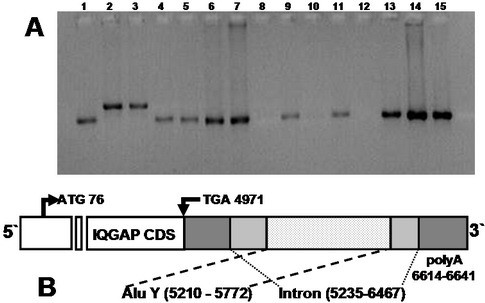
**(a) AL040372-specific fragments in a variety of primates**. The arrow indicates the increase of the fragment size in *Ateles *and *Callimico *due to Alu insertion. Lanes: 1, *Lemur*, 2, *Ateles *(Alu insertion); 3, *Callimico *(Alu insertion); 4, *Colobus; *5, *Erythrocebus; *6, *Cercopithecus; *7, *Macaca; *8, *Hylobates; *9, *Pongo *(Sumatran); 10, *Pongo *(Bornean); 11, *Gorilla *(sample 1); 12, *Gorilla *(sample 2); 13, *Pan *(sample 1); 14, *Pan *(sample 2); 15, *Homo sapiens*. **(b) **Localization of Alu sequences in the IQGAP gene (UniGene cluster Hs. 133294).

In some primates (*Callimico goeldii, Ateles fusciceps*), Alu sequences were found in AL040372-homologous fragments (Fig. 2a, lanes 2 and 3). These Alu sequences belong to type Y, as shown by sequencing (data not shown). Fig. 2b shows the location of the insert on the genetic map of the IQGAP gene.

## Discussion

The prediction that evolutionarily new sequences may be expressed in tumor cells was made in our previous articles [1,2]. To experimentally examine this prediction, we performed Southern hybridization of [α-^32^P]-labeled newly described tumor-related fragments with genomic DNA from different animal species. Sequences studied in the present article were selected from tumor-related transcripts revealed by an *in silico *search and experimentally described in our previous papers [3,4].

Hybridization signals were detected only with human and orangutan DNA, with the single exception of a signal observed after hybridization of the AA166653-specific [α-^32^P]-labeled probe with chicken DNA [see Additional file 1]. This signal was consistently observed in several hybridization experiments. However, comparative genomics analysis has not revealed AA166653-homologous sequences in the chicken genome. We suggest that this signal may be an artifact of hybridization.

Interestingly, in the case of the AA166653-homologous sequence, signals on Southern blot form a "ladder" [see Additional file 1], which is a feature of fragments located in a repetitive sequence. It is in good agreement with computational evidence that the AA166653-specific sequence is located in an intergenic spacer upstream of the 23 repeat region of the human ribosomal DNA complete repeating unit [4], which is tandemly repeated and forms arrays in genomes of eukaryotes.

Comparative genomics analysis have shown that the tumor-related transcripts under consideration have orthologs in mammal genomes only and not in those of fishes, amphibia, and birds, with the single exception of a short sequence in the chicken genome with low homology for AI952931 (Tables 2 and S1).

The reason why the probe did not hybridize with DNA from mammals in which we found homologous sequences using comparative genomics analysis is due to low homology and the short length of orthologous sequences (Table 2).

We may conclude that Southern hybridyzation and comparative genomics data confirm the evolutionary novelty of the sequences studied, i.e., their origins in mammals or in primates.

The results of molecular phylogenetic analysis are in accordance with Southern hybridization and comparative genomics results. AA166653-homologous sequences are present only in apes and macaques and have no homology with any sequences in other mammals. The most archaic of the four species presented on the phylogenetic tree in Fig. 1b is the macaque. We cannot find an AA166653-specific sequence in primates before the divergence of old world monkeys and apes. Therefore, the origin of AA166653-specific sequences took place about 25 mya, during the divergence of macaques and apes.

AL040372-, AI792557- and AI952931-specific sequences formed separate clusters on phylogenetic trees demonstrating high nucleotide sequence divergence (from 20% to 35%) with related sequences in mammals (Fig. 1a,1c and 1d). AL040372-homologous sequences were found in lemurs – the most archaic members of the primate group. Lemur sequences demonstrate lower divergence from other primates (about 8%) than related sequences from non-primate animals (20% and more). Phylogenetic analysis has shown that lemur sequences belong to the primate phylogenetic cluster. Other primates form a separate non-lemur subcluster in this phylogenetic cluster (Fig. 1a).

AI792557-homologous sequences form a well-supported monophyletic group in apes and old world monkeys. These sequence homologs were found in the *Ateles-Callimico *group and were not present in older primates. The divergence of the *Ateles-Callimico *group from old world monkeys took place about 40 mya The cDNA of Hs.133107 which includes EST AI952931, is identified as PVT1, encoding for the Pvt1 oncogene homolog. The Pvt1 locus also is a common integration site for murine leukemia viruses on mouse chromosome 15 and is located approximately 270 kb from *c-myc*. MLV proviruses integrated in the Pvt1 locus activate *c-myc *expression by long-range (up to-300 kb) *cis*-effects [6]. In the human genome, the corresponding sequence is located on chromosome 8. Therefore, an evolutionarily new tumor-specific sequence with a high potential of oncogenicity is presented in the mammalian lineage near Pvt1 locus. Obvious overexpression of AI792557-specific transcripts in human tumors [3,4] could be explained by enhanced transcriptional activity of the *c*-*myc*-regulating element.

The proportion of the mammalian genome which is transcribed is greater than usually realized [7,8]. It turns out that large regions of the genome beyond the coding segments are transcribed, producing non-coding RNAs [3,7,9]. As shown in this article two of ESTs studied are from introns (plus or minus chains), one from intergenic spacer region and one represent 3-UTR of mRNA, containing alternativerly spliced intron. According to our previous data [4], they do not contain easily recognized open reading frames or contain only short open reading frames.

There is a growing number of recent publications on non-coding RNAs and their possible functions [10-12]. But the fact that certain RNAs have low coding potential may also characterize them as evolving sequences. The concept of evolution by gene duplication [13] involves understanding that the extra copy of the duplicated gene may accumulate mutations and acquire a new function. Before acquisition of a new function, it may express RNA without long open reading frames or with stop-codons and/or frame-shift mutations interrupting open reading frames. In the similar way, non-coding sequences could evolve and eventually acquire a function and/or longer open reading frames. The fact that we were able to demonstrate duplications of AL040372- and AA166653-homologous sequences in the human, chimpanzee, and macaque genomes (Table 3) supports this interpretation.

The Alu-Y element was found in a AL040372-homologous sequence in *Ateles *and *Callimico*. The presence of the Alu sequence in the genome may mediate DNA recombination, the creation of new exons, and the donation of new regulatory elements [14]. It was found in our study that part of the AL040372-homologous sequence in the lemur genome has an extension with no similarity in those of other primates (data not shown). In higher primates, this region demonstrates a homology with the human genome.

Taken together, these data from Southern hybridization experiments, molecular phylogenetic studies, and computational evidence suggest that AA166653-, AL040372-, AI792557- and AI952931-homologous sequences are indeed evolutionarily new. They originate in mammals (AA166653 – in primates) and form phylogenetic clusters in primates. They are not expressed in normal cells [3,4], i.e., they are sleeping.

Earlier [1,2], we formulated the concept of the positive evolutionary role of tumors. According to this concept, tumors provide conditions for the expression of evolutionarily new and/or sleeping genes in their cells. As evolutionary new genes we defined genes which participate in the origin of new cell types [2]. New cell type origin is very rare event which is associated with progressive evolution. During 10^9 ^years of multicellular organisms evolution only about 200 specialized cell types have been originated [2]. Thus, within the framework of our hypothesis sequences originated in mammalas may be well considered as evolutionary new.

We may guess that during the earliest period of the origin of mammals, genome evolution and cellular proliferative tumor-like processes provided material for the origin of diversity of mammalian cell and tissue types by generating a diversity of new gene expression patterns. Populations of tumor-bearing animals could be ancestors of the first mammals. Present-day tumors (at the earlier stages of progression) may somehow recapitulate these processes.

## Conclusion

Our data presented in this and previous articles [3,4] demonstrate the expression of relatively evolutionarily new (in respect to progressive evolution) and/or sleeping sequences in tumor cells and support the concept of the possible evolutionary role of tumors as a proving ground or evolutionary reservoir of expression. If proven to be correct, this concept may substantially increase our capabilities in the diagnosis and treatment of cancer. This concept may also describe one of the mechanisms of progressive evolution of animal species in which tumors participate.

## Methods

### Genomic DNA

Human, ape (Pan troglodytes, Gorilla gorilla, Pongo pygmaeus, Hylobates concolor), old world monkey (Erythrocebus patas, Macaca mulatta, Colobus guereza, Cercopithecus aethiops), and new world monkey (Callimico goeldii, Lemur catta, Ateles fusciceps) genomic DNAs were used in the study. All samples except human DNA were kindly provided by Dr. S. O'Brien (Chief, Laboratory of Genomic Diversity, National Cancer Institute). The DNA concentration of each sample was brought to 200 ng/μl before being used.

### PCR analysis

Oligonucleotide primers for PCR were designed with OLIGONEW software after alignment of human EST sequences and the corresponding regions of the human genome. We performed BLAST searches for all primer pairs created. Only PCR primers that corresponded to a unique location in the human genome and to an EST cluster of interest were used.

Primers for AA166653: 5'-TCTTTCTTGATGAATTATCTTATG-3' and 5'-ACACACCCTCATTCCCGC-3'; the expected fragment size is 443 bp. Primers for AL040372: 5'-GTCAACCTTCTCATCTTCCTC-3' and 5'-CAGGAAGTTGGGTAGATGTG-3'; the expected fragment sizes are 412 bp on cDNA and 1084 bp on genomic DNA. Primers for AI952931: 5'-TAATTGCATTCTTCAAAATTCTAC-3' and 5'-CTTCGCACCATTGAATAAAC-3'; the expected fragment size is 315 bp. Primers for AI792557: 5'-TACATAGTTGTTATCTTAAGGTG-3' and 5'-TGGGAATTCTATACTTTTGAC-3'; the expected fragment size is 344 bp. Histone H4 control primers: 5'-ATGTCTGGCCGTGGTAAAGG-3' and 5'-CCGAAGCCGTAAAGAGTGCG-3'; the expected fragment size is 300 bp.

The PCR mixture contained 500 ng of genomic DNA as template, PCR buffer (1), MgC12 (4 mM), dNTP (each at 200 μM), specific forward and reverse primers (each at 0.2 μM), and Taq DNA Polymerase (1 u) in a total volume of 25 μl (all reagents were supplied by Fermentas, Lithuania).

PCR was carried out under the following conditions: 1 min at 95°C, 35 cycles each consisting of 30 s at 95°C and 30 s at 56°C for AA166653 primers and histone H4 primers or at 58°C for all other primers, and 1 min at 72°C. At the final stage of the PCR reaction, mixtures were incubated for 5 min at 72°C to elongate the DNA fragments synthesized. PCR products were separated by electrophoresis in 2% agarose gel and visualized by staining with ethidium bromide.

### Southern hybridization

DNA samples were digested with HindIII (10 U per μg of DNA) for 16 h at 37°C. Digested DNA (8 μg per lane) was electrophoresed in 0.8% agarose gel overnight at 25 V/cm. Gels were stained with ethidium bromide to assess loading and blotted onto a nylon membrane, Hybond-N (Amersham, USA), according to the manufacturer's instructions.

PCR products specific for genes of interest were labeled with [α-^32^P]dCTP using the HexaLabel DNA Labeling Kit (Fermentas, Lithuania) according to the manufacturer's instructions. Filter prehybridization and hybridization were carried out according to the standard procedure [15]. Washing conditions were as follows: two times in 0.25 M sodium phosphate (pH 7.2), 5% SDS for 30–60 min at 65°C and two times in 0.125 M sodium phosphate (pH 7.2), 1% SDS for 30–60 min at 65°C (medium stringency) or two times in 20 mM sodium phosphate (pH 7.2), 1% SDS for 30–60 min at 65°C (high stringency). X-ray films were exposed to the blots for 3 days at -70°C with an intensifying screen.

### Cloning and sequencing

Amplified fragments were cloned by standard techniques using the bacterial plasmid vector pGEM-T Easy (Promega, USA). Colonies of recombinant DH10B/R *E. coli *cells obtained by electrotransformation were selected. We subjected recombinant plasmids to restriction endonuclease analysis and isolated those with fragments of interest using the Wizard Minipreps Plasmid DNA Purification System (Promega, USA). Multiple clone sequencing was performed for each amplicon.

Sequencing was carried out by the Sanger method using the AutoCycle Sequencing Kit (Pharmacia Biotech, Sweden) and standard Cy5-labeled primers T7, whose binding sites flank the cloning site of recombinant fragment. We analyzed the products of the sequence reaction with an automated sequencer, ALFexpress (Pharmacia Biotech, Sweden), using the ALFwin v. 1.10 software package (Pharmacia Biotech, Sweden).

### Molecular phylogenetic analysis

PCR amplified fragments of primate DNA were cloned as described above. A plasmid collection from each primate was created. In total, 86 clones containing sequences of interest were obtained. For each fragment, at least two clones were sequenced in forward and reverse directions in order to exclude PCR and sequencing errors. The BioEdit software was used to generate sequence alignments. The alignments consist of the following numbers of phylogenetically informative sites: 412 for the AL040372 fragment, 443 for the AA166653 fragment, 315 for the AI952931 fragment, and 344 for the AI792557 fragment. We constructed phylogenetic trees using the neighbor-joining method. Distance-based reconstructions and parsimony reconstructions based on the optimal alignments gave qualitatively similar phylogenetic results, with the same major clades and topological differences in nodes. The results of phylogenetic analysis are presented in Fig. 1.

Sequencing data were analyzed with the DNASIS v. 2.5 software (Hitachi Software Engineering, USA). We carried out alignments using the BioEdit software and excluded gap-containing sites. Phylogenetic trees were built according to the neighbor-joining method using the Kimura distances by the DNADIST and NEIGHBOR modules of the PHYLIP software package and PHYLIP v.3.57c [16], respectively. The reliability of the tree topology was assessed by bootstrapping with 1,000 replicates (the SEQBOOT and CONSENCE modules of the PHYLIP). The tree was drawn with Tree View software.

### Identification of gene duplications and comparative genomics analysis

BLAT searches among primate genome nucleotide sequences were conducted to reveal duplications of sequences under analysis. Matches with a level of identity greater than or equal to 80% of maximum for each sequence were taken as duplications.

The cross-species chained alignments database integrated in the Genome Browser tool was used to search for orthologous sequences in fugu, tetraodon, zebrafish, frog, chicken, rat, mouse, cow, dog, macaque, and chimpanzee genomes [17].

## Competing interests

The author(s) declare that they have no competing interests.

## Authors' contributions

A.P.K. is an author of original hypothesis of the evolutionary role of tumor and general design of experiments. He also directed the whole research. D.P and L.L.K. performed PCR and Southern experiments. I.D. carried out sequencing and molecular phylogenetic analysis. Y.G. and N.S. conducted comparative genomics and bioinformatics analyses. A.B. participated in the intial stage of bioinformatics analysis. All authors discussed the results and commented on the manuscript.

## Supplementary Material

Additional file 1 **Supplementary Information**. The file contains details about Southern hybridization analysis results (Figures S1 and S2). Click here for file

Additional file 2 **Supplementary Information**. **Table S1. **Cross-species alignment results for sequences within experimentally analyzed PCR fragments. **Figures S3a, S4a, S5a, and S6a **show BioEdit alignments of human genome sequences corresponding to fragments of interest with homologous sequences in the mouse and chimpanzee genomes. In Fig. S6a, homologs from cow and dog genomes are added. Primers that had been used for RT-PCR experiments are shown. The graphical alignments of the ESTs under study (set in the light yellow box in the pictures) with the human genome are present in **Figures S3b, S4b, S5b, and S6b**. Alignments of human genome fragments with corresponding genome fragments of other organisms (fugu, tetraodon, zebrafish, frog, chicken). Click here for file
