# Supplementary material for: Evolutionarily new sequences expressed in tumors
Source: Infect Agent Cancer. 2006 Dec 25;1:8. doi: 10.1186/1750-9378-1-8 (PMC1779766; doi:10.1186/1750-9378-1-8)
Supplement: Additional file 1 — Supplementary Information. The file contains details about Southern hybridization analysis results (Figures S1 and S2). [file 1750-9378-1-8-S1.doc]

## Results of Southern hybridization analysis (Figures S1 and S2)

We studied sequences from UniGene clusters Hs.133107 (AI792557), Hs.426704 (AA166653), Hs.128594 (AI952931), and Hs.133294 (AL040372) which according to our computational and experimental analysis were characterized as tumor-related*^3,4^*. We performed Southern hybridization of [α-^32^P-labeled sequence-specific fragments with genomic DNA from eleven different animal species: lamprey, fish, frog, chicken, pigeon, mouse, rat, guinea pig, sheep, horse, and human.

Southern hybridization of a 443-bp AA166653-specific [α-^32^P-labeled fragment with genomic DNA from different animal species (Fig. S1a) results in two major (11.7-kb and 9.5-kb) and two minor (14.6-kb and 2.0-kb) bands in human DNA and a signal corresponding to a 13.7-kb band in chicken DNA. No hybridization signal was detected in lamprey, fish, frog, pigeon, mouse, rat, guinea pig, sheep, and horse DNA. Southern hybridization of a 315-bp AI952931-specific labeled fragment produced signals only with human DNA–two strong bands corresponding to 10.9 kb and 8.6 kb and a weak band corresponding to 1 kb (Fig. S1b). Southern hybridization of a 344-bp AI792557-specific labeled fragment resulted in hybridization signals with human DNA only (Fig. S1c): bands corresponding to sizes of 8.2 kb, 6.5 kb, and 1.9 kb were detected. It should be noted that in the case of this sequence the quality of the Southern blots was always poor regardless of differences in probes or human DNA samples. Southern hybridization of a 1084-bp AL040372-specific labeled fragment with genomic DNA from different animal species revealed 13.6-kb, 3.1-kb, and 1.9-kb fragments hybridizing to the probe in human DNA only (Fig. S1d). PCR experiments with primers for the histon H4 gene on a panel of DNAs from different species show that all DNAs used in this study are reactive with primers of an evolutionarily conserved gene: a major band corresponding to 300 bp was produced (Fig. S1e). The weaker intensity of this band in birds (chicken and pigeon) may be due to the smaller concentration of corresponding DNAs in the PCR experiment. Similar hybridization results were obtained both with medium and with high stringency washings.

In addition, we were able to demonstrate by Southern hybridization that a sequence homologous to the human AA166653-specific 11.2-kb fragment is present in orangutan DNA (Fig. S2).


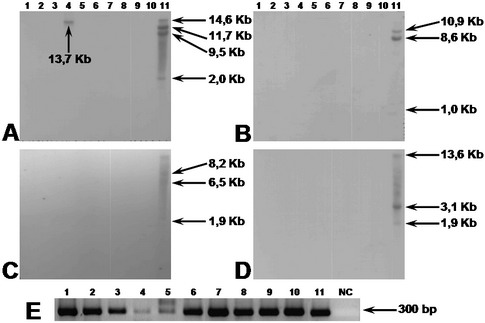


## Figure S1. Southern hybridization of [α-32P]-labeled fragments with Hind III-digested genomic DNA from different animal species

1. AA166653-specific fragment;
2. AI952931-specific fragment;
3. AI792557-specific fragment;
4. AL040372-specific fragment;
5. PCR amplification of the fragment of histon gene H4 on genomic DNA from different animal species (NC, negative control). Lanes: 1-lamprey; 2-fish; 3-frog; 4-chicken; 5-pigeon; 6-mouse; 7-rat; 8-guinea pig; 9-sheep; 10-horse; 11-human.


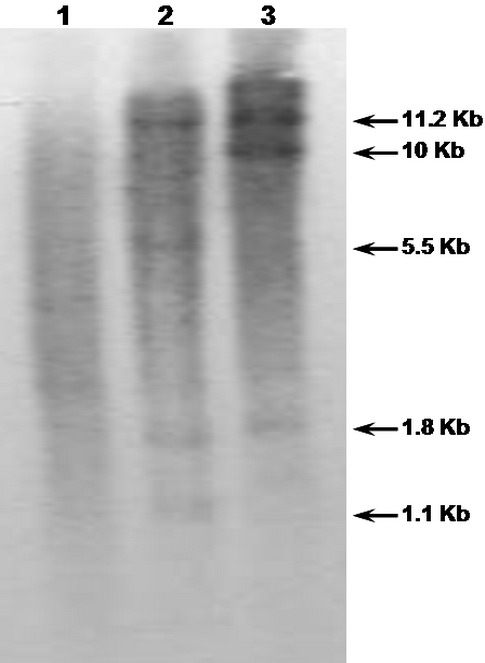


## Figure S2. Southern hybridization of the AA166653-specific fragment with genomic DNA from different primate species (Hind III).

Lanes: 1, baboon; 2, orangutan; 3, human.
