# Supplementary material for: Evolutionarily new sequences expressed in tumors
Source: Infect Agent Cancer. 2006 Dec 25;1:8. doi: 10.1186/1750-9378-1-8 (PMC1779766; doi:10.1186/1750-9378-1-8)

## Table S1. Cross-species alignment results for sequences within experimentally analyzed PCR fragments.

| Sequence/Cluster | Human Position | Compared Genomes where Homology was found* | Aligned Bases between Genomes | % of Aligned Bases** | Matched Bases between Genomes | % of Matched Bases*** | Human Sequence | | Compared Sequence | |
| --- | --- | --- | --- | --- | --- | --- | --- | --- | --- | --- |
|  |  |  |  |  |  |  | Full length of Aligned Sequence | Unmatched Bases | Full length of Aligned Sequence | Unmatched Bases |
| [GenBank:AL040372]/133294 | chr1:153 308 314-153 309 398 | opossum | 963 | **88.8** | 504 | **52.3** | 1085 | 581 | 2066 | 1562 |
|  |  | mouse | 874 | **80.6** | 536 | **61.3** |  | 549 | 769 | 233 |
|  |  | rat | 1009 | **93.0** | 620 | **61.4** |  | 465 | 893 | 273 |
|  |  | cow | 962 | **88.7** | 702 | **73.0** |  | 383 | 910 | 208 |
|  |  | dog | 1085 | **100.0** | 712 | **65.6** |  | 373 | 1227 | 515 |
|  |  | rhesus | 1084 | **99.9** | 1017 | **93.8** |  | 68 | 1071 | 54 |
|  |  | chimpanzee | 1084 | **99.9** | 1069 | **98.6** |  | 16 | 1075 | 6 |
| [GenBank:AI792557]/133107 | chr8:129 160 449-129 160 793 | mouse | 340 | **98.6** | 201 | **59.1** | 345 | 144 | 492 | 291 |
|  |  | rat | 343 | **99.4** | 203 | **59.2** |  | 142 | 479 | 276 |
|  |  | cow | 344 | **99.7** | 231 | **67.2** |  | 114 | 335 | 104 |
|  |  | dog | 340 | **98.6** | 251 | **73.8** |  | 94 | 550 | 299 |
|  |  | rhesus | 344 | **99.7** | 327 | **95.1** |  | 18 | 342 | 15 |
|  |  | chimpanzee | 344 | **99.7** | 339 | **98.5** |  | 6 | 340 | 1 |
| [GenBank:AA166653]/426704 | chr2:132 864 460-132 864 902 | rhesus | 386 | **87.1** | 354 | **91.7** | 443 | 89 | 488 | 134 |
|  |  | chimpanzee | 442 | **99.8** | 440 | **99.5** |  | 3 | 445 | 5 |
| [GenBank:AI952931]/128594 | chr3:54 641 214-54 641 528 | chicken | 170 | **54.0** | 92 | **54.1** | 315 | 223 | 502 | 410 |
|  |  | opossum | 200 | **63.5** | 134 | **67.0** |  | 181 | 206 | 72 |
|  |  | mouse | 314 | **99.7** | 204 | **65.0** |  | 111 | 305 | 101 |
|  |  | rat | 314 | **99.7** | 205 | **65.3** |  | 110 | 300 | 95 |
|  |  | cow | 314 | **99.7** | 243 | **77.4** |  | 72 | 318 | 75 |
|  |  | dog | 314 | **99.7** | 253 | **80.6** |  | 62 | 320 | 67 |
|  |  | rhesus | 314 | **99.7** | 292 | **93.0** |  | 23 | 324 | 32 |
|  |  | chimpanzee | 314 | **99.7** | 312 | **99.4** |  | 3 | 315 | 3 |

* Fugu, tetraodon, zebrafish, frog, chicken, rat, mouse, cow, dog, macaque, and chimpanzee genomes were analyzed.

** Percent of aligned bases were estimated as the ratio of aligned bases between genomes and the full length of the aligned human sequence.

*** Percent of matched bases were estimated as the ratio of matched and aligned bases between genomes.

Figures S3a, S4a, S5a, and S6a show BioEdit alignments of human genome sequences corresponding to fragments of interest with homologous sequences in the mouse and chimpanzee genomes. In Fig. S6a, homologs from cow and dog genomes are added. Primers that had been used for RT-PCR experiments are shown.

The graphical alignments of the ESTs under study (set in the light yellow box in the pictures) with the human genome are present in **Figures S3b, S4b, S5b, and S6b**. Alignments of human genome fragments with corresponding genome fragments of other organisms (fugu, tetraodon, zebrafish, frog, chicken, rat, mouse, cow, dog, macaque, and chimpanzee) are shown. The aligned sequences of other species represent possible orthologs for the fragments under study.

## Fig S3a. HS.133107 H. sapiens genome region: chr8:129160366-129160862 + strand

10 20 30 40 50 60 70 80 90 100

....|....|....|....|....|....|....|....|....|....|....|....|....|....|....|....|....|....|....|....|

**H.sapiens**  **ccaccacacttggctagttagggtttttttgtTTTGTTTTGTTTTTGGGttttttccttttcttttctttttttttttttttttACATAGTTGTTATCTT**

**AI792557**  **--------------------------A.GATCGC.CGACATCGA.AC.AC................................--................**

**fwd primer**  **-----------------------------------------------------------------------------------.................**

**rev primer**  **----------------------------------------------------------------------------------------------------**

**P.troglodytes** **----------------------------------------------------...TTCC..T.C..T.C...g......................-----**

**M.musculus**  **------------------------------------------------------------------------------..cccCCaTccC.....T...g**

110 120 130 140 150 160 170 180 190 200

....|....|....|....|....|....|....|....|....|....|....|....|....|....|....|....|....|....|....|....|

**H.sapiens**  **AAGGTGATTTCCAATTTTTTTTTCCATTTACATTTTTCCACAAGCATTGTCCACTTTATTCTGTAACCTTTTCAACTACCATTTTGAAATTTGCTTTTAT**

**AI792557**  **......................-.............................................................................**

**fwd primer**  **......------------**

**rev primer**  **----------------------------------------------------------------------------------------------------**

**P.troglodytes** **.............................................................c...C.A....-------------...............**

**M.musculus**  **----------------------------------------.c....a..c.tggg......ca...-...g........-..g.........t.......**

210 220 230 240 250 260 270 280 290 300

....|....|....|....|....|....|....|....|....|....|....|....|....|....|....|....|....|....|....|....|

**H.sapiens**  **CCATGTGGTTGTTTGTGATGAACTACAGGTTGCTGA--CTTTCTTCCCCTTCT-----GTAAATAAAGTTTTCTTCCTAGTATGTCCTGTATCTCAAGAG**

**AI792557**  **....................................--...............-----..........................................**

**fwd primer**

**rev primer**  **----------------------------------------------------------------------------------------------------**

**P.troglodytes** **....................................--...............-----..........................................**

**M.musculus**  **...aa.tc.....a.ca.aa..a.C.gtAa.TTctTag....t.ctt.....ctgtaT.....a...a.....c.t....a....t..a..ctca..act**

310 320 330 340 350 360 370 380 390 400

....|....|....|....|....|....|....|....|....|....|....|....|....|....|....|....|....|....|....|....|

**H.sapiens**  **GATCTCATCAGTGGAATCATTAGATCAAAGGATATGACTGTTGCTCAGCTCTCTGTGTGTATGTaaattaatagg-----ctgtttatttgagcagttgt**

**AI792557**  **...........................................................................-----....................**

**fwd primer**

**rev primer**  **----------------------------------------------------------------------------------------------------**

**P.troglodytes** **...........................................................................-----....................**

**M.musculus**  **c..ga..ga...ct.gc................g...a.....tcacag.g.a..a....t...gtt.....ta.Tgaacg.....tcc...at..c.a.**

410 420 430 440 450 460 470 480 490 500

....|....|....|....|....|....|....|....|....|....|....|....|....|....|....|....|....|....|....|....|

**H.sapiens**  **aggcttacaaaaatattgagtcaaaagtatagaattcccatatattctcctcttctcccgtcatatcccctattattcgcatcttgaattaatatggtac**

**AI792557**  **...........................................................T..TGTCGT.T.C.CGC**

**fwd primer**

**rev primer**  **-------------------.....................**

**P.troglodytes** **...........................................................**

**M.musculus**  **..at...t..gg.a..cc.c.g...gc.gc.c.c.agtggc.c..gtctt.aa..c.agcattctgat.tcTGAG..tAAgAgca.tc.ggtcTAcA**

....|....

**H.sapiens**  **attttttac**

**AI792557**

**fwd primer**

**rev primer**

**P.troglodytes**

**M.musculus**

## Fig S3b H. sapiens genome region: chr8:129160366-129160862


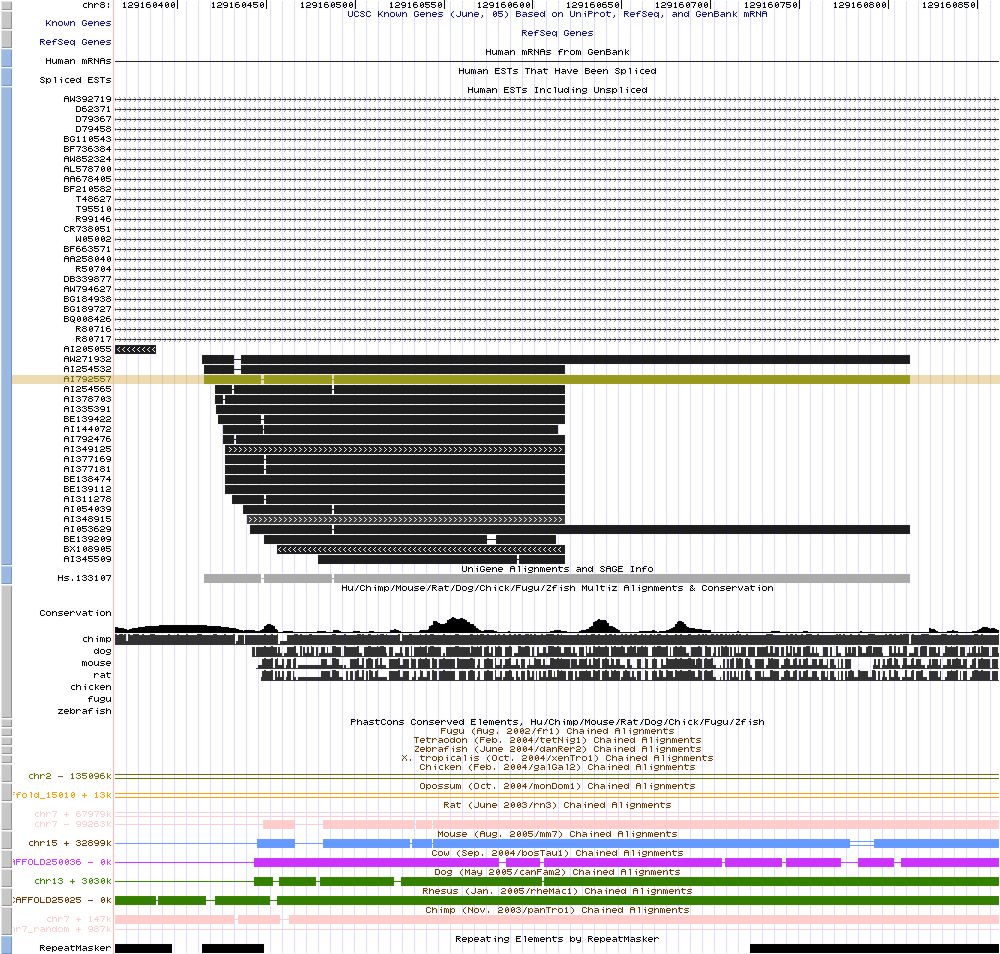


## Fig S4a. HS.133294 H. sapiens genome region: chr1:153308314-153309400 + strand

10 20 30 40 50 60 70 80 90 100

....|....|....|....|....|....|....|....|....|....|....|....|....|....|....|....|....|....|....|....|

**H.sapiens**  **---------------TGTGGGAGTAACAGCTGCTTTTATTAACATCAGAAGGGCAACAGTACAGGAAGTTGGGTAGATGTGGGGACAACAGAGAGACTGT**

**AL040372**  **-G-----------------............................T....................................................**

**fwd primer**  **----------------------------------------------------------------------------------------------------**

**rev primer**  **-------------------------------------------------------------....................**

**P.troglodytes** **---------------....................................................A................................**

**M.musculus**  **------------------..A.A.....-----.........A...TAG......GTGAGG.......A....-...........GG.......-----.**

110 120 130 140 150 160 170 180 190 200

....|....|....|....|....|....|....|....|....|....|....|....|....|....|....|....|....|....|....|....|

**H.sapiens**  **GGCAGAGGCAGGACTGCAGATCTATGGAAATTGCCTGGAAGAGTCAGCTGTAAGGGATGAGAATCCTGAGGGTAAAAGAGAAAAGGGAAAGACTCCTCTT**

**AL040372**  **..................A...............................................----------------------------------**

**fwd primer**  **----------------------------------------------------------------------------------------------------**

**rev primer**

**P.troglodytes** **............................................................................--......................**

**M.musculus**  **...CA....T..C..A...G......AG..CCA........--......---------------------------------------------------**

210 220 230 240 250 260 270 280 290 300

....|....|....|....|....|....|....|....|....|....|....|....|....|....|....|....|....|....|....|....|

**H.sapiens**  **TGATCTTATGAAGCTGAAATAACAAGATCTTAAACATGAGTGAGAATCTGTTGCCCCAACCTAAGGTGACTTTAAATCCAAGGTAAAAAACACGGCATGG**

**AL040372**  **----------------------------------------------------------------------------------------------------**

**fwd primer**  **----------------------------------------------------------------------------------------------------**

**rev primer**

**P.troglodytes** **....................................................................................................**

**M.musculus**  **------------------------------------------------------------..G.......---------------G.G..G.T....---**

310 320 330 340 350 360 370 380 390 400

....|....|....|....|....|....|....|....|....|....|....|....|....|....|....|....|....|....|....|....|

**H.sapiens**  **GTATTAGTTTGAATAGGGAAAATGAGAACTCTCTTTGAGCTCAAAAAAAAAAAAAAAAAAAAAAAAATGAAAGCGTTAAAACCCTGATTAAGTCTGCACA**

**AL040372**  **----------------------------------------------------------------------------------------------------**

**fwd primer**  **----------------------------------------------------------------------------------------------------**

**rev primer**

**P.troglodytes** **..........................................---------.................................................**

**M.musculus**  **-------------------.G.CA........----...T...G...GTG.G..---------------------........A........G.CA....**

410 420 430 440 450 460 470 480 490 500

....|....|....|....|....|....|....|....|....|....|....|....|....|....|....|....|....|....|....|....|

**H.sapiens**  **ATGATCCAGAGTGTAAGGATGGGAGAAAGAATAAATACCCTAGTGACCCACATATTAAACAGACCACAGACAAGAGAACAAGACTTGAAGCTAATGGAAG**

**AL040372**  **----------------------------------------------------------------------------------------------------**

**fwd primer**  **----------------------------------------------------------------------------------------------------**

**rev primer**

**P.troglodytes** **.................................G..................................................................**

**M.musculus**  **.CA...---------...T.-------.............C.............AG..C..--.....AGTGG.GA..GG...T....G.----------**

510 520 530 540 550 560 570 580 590 600

....|....|....|....|....|....|....|....|....|....|....|....|....|....|....|....|....|....|....|....|

**H.sapiens**  **GTCATCTTGCCCATGCCGCCATGGGGGGCAACAGTGCCACAATGCCACATGGGCACTAACACACACTGCATCCCCCCAGTCCCTGCCCAGGTTGGAGGGT**

**AL040372**  **----------------------------------------------------------------------------------------------------**

**fwd primer**  **----------------------------------------------------------------------------------------------------**

**rev primer**

**P.troglodytes** **....................................................................................................**

**M.musculus**  **-------..TG..CT.A..TG.-------------------------.....A..TC.G..AGTG.....C.AAGT..AGG.TA.GA....A..AGA...**

610 620 630 640 650 660 670 680 690 700

....|....|....|....|....|....|....|....|....|....|....|....|....|....|....|....|....|....|....|....|

**H.sapiens**  **GTGCAGATCACAGCAGCAGAGCTGCCTAGACTCAGGAAGGGCAGGAACACCCACTGCTGATGCAGTGGGAGGTAGGGATGGGGAGCCTGGCCCTGGCTTT**

**AL040372**  **----------------------------------------------------------------------------------------------------**

**fwd primer**  **----------------------------------------------------------------------------------------------------**

**rev primer**

**P.troglodytes** **....................................C...............................................................**

**M.musculus**  **...AG.GG.......-.....GGA...G.G..T...--..A...CG.......TG...T...A..C..-..T.G..C.--.AC..........G......**

710 720 730 740 750 760 770 780 790 800

....|....|....|....|....|....|....|....|....|....|....|....|....|....|....|....|....|....|....|....|

**H.sapiens**  **GTGGGAGTGCAGAGAGAAGGAAGGCAGAGGGGAAATCAAGCCGCTGGGGCAGTGCTTGTAATATTGGGGTGACTGTGGGAGGGCAGTAGCAGACACAAGA**

**AL040372**  **----------------------------------------------------------------------------------------------------**

**fwd primer**  **----------------------------------------------------------------------------------------------------**

**rev primer**

**P.troglodytes** **....................................................................................................**

**M.musculus**  **.GAA.CCC.TG..A..----------..A....G..A.G..T......-.......A.G.CG.C.........CACAC...A........-.......A.**

810 820 830 840 850 860 870 880 890 900

....|....|....|....|....|....|....|....|....|....|....|....|....|....|....|....|....|....|....|....|

**H.sapiens**  **GTAATGGCTTTCCCAGGTCAAGGTCCATGTCCTACCATCTGGCAGGAAAGCCAGGGGTTTGTCATGCATGATAAAAGCCACACAGCTGGACTCTGGGCAA**

**AL040372**  **------------------------------------................................................................**

**fwd primer**  **----------------------------------------------------------------------------------------------------**

**rev primer**

**P.troglodytes** **...................................................................................G................**

**M.musculus**  **CC.TG.TTC..TG....A.TGA..G..G.CT.------....T------T..C....A.--------G..G..........C.G..C....CTG.A...T**

910 920 930 940 950 960 970 980 990 1000

....|....|....|....|....|....|....|....|....|....|....|....|....|....|....|....|....|....|....|....|

**H.sapiens**  **GGCCCACTTTAGCCAATTAGAAGATACACTGTCTGTGGCCAGGCAGGCAAGCTCCTCCCAGCTGGGGAAGGGGTGAGAATCCCTGGGCCTTGCCCAGTCC**

**AL040372**  **.........................................................................G..........................**

**fwd primer**  **----------------------------------------------------------------------------------------------------**

**rev primer**

**P.troglodytes** **....................................................................................................**

**M.musculus**  **.........C......--------------------------.T...AC.AT.....TTG.GC...--....TCA..C..TGTG.-------------..**

1010 1020 1030 1040 1050 1060 1070 1080 1090 1100

....|....|....|....|....|....|....|....|....|....|....|....|....|....|....|....|....|....|....|....|

**H.sapiens**  **TGAGCTCTAGGTGTCTGCAGGGAAGCACAGTGGTGAGTTAGTGTTAAAGAAAGCATCCAGAGAGGTAAGAGGGGCTTGGGTAGCACCCTTTGCCTCTGTC**

**AL040372**  **........-..................................G......................................................-.**

**fwd primer**  **----------------------------------------------------------------------------------------------------**

**rev primer**

**P.troglodytes** **........G...........................................................................................**

**M.musculus**  **C.......G.---...T.G.A...A---..A.C..G..A.TG.AG..GCGC....G....TC..C.--...A...C.CA..TC.G..---...A..C...**

1110 1120 1130 1140

....|....|....|....|....|....|....|....|....|...

**H.sapiens**  **ACTTCCGCAAAAACTTCTTGTTGAGGAGGAAGATGAGAAGGTTGACAT**

**AL040372**  **................................................**

**fwd primer**  **-------------------------.....................**

**rev primer**

**P.troglodytes** **................................................**

**M.musculus**  **.......A.GG..............C...........G..A.....G.**

## Fig S4b. H. sapiens genome region: chr1:153308314-153309400


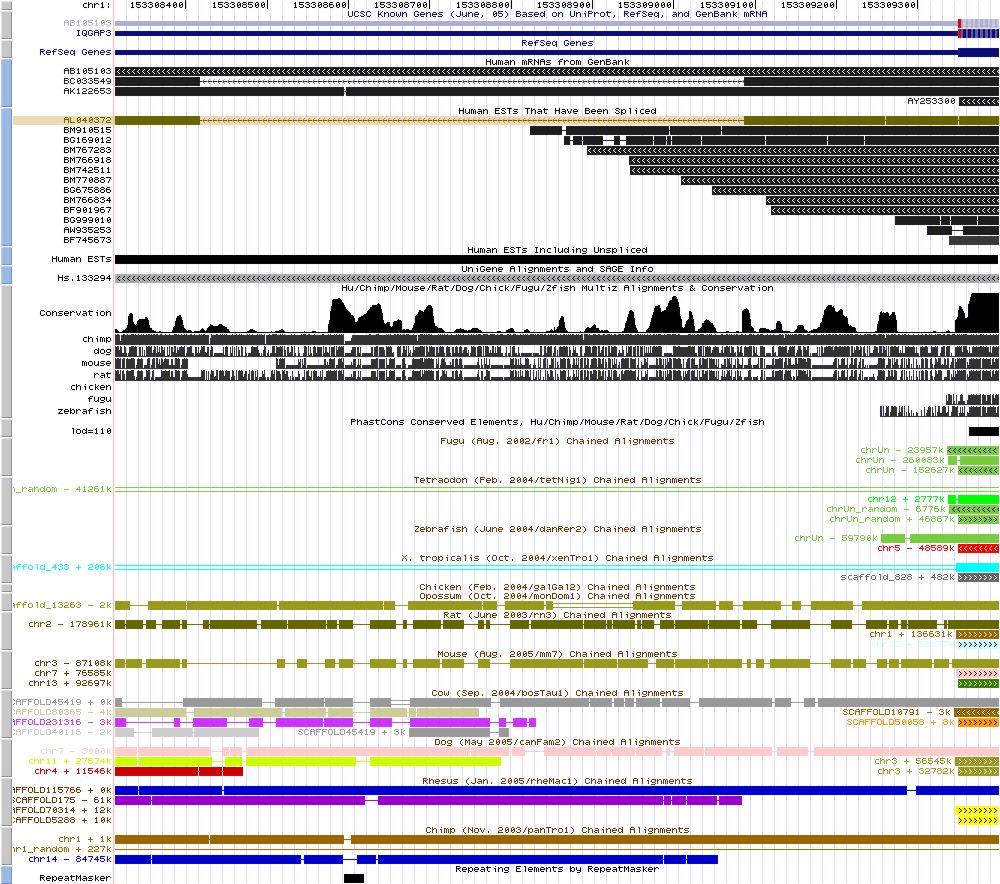


Fig S5a. HS.426704 H. sapiens genome region: chr2:132864310-132864909 - strand

10 20 30 40 50 60 70 80 90 100

....|....|....|....|....|....|....|....|....|....|....|....|....|....|....|....|....|....|....|....|

**H.sapiens**  **TTCttatttattgatgaattattttcttatttatatttgtttatttatttTCAGACCGAGTCTTGCTCTGGGCGAGGCGAGGAGGGGCG-----------**

**AA166653**  **------...c............c..a.g......t.g...--.c............g...n-.c..........g.......c.a....aggcacagcgc**

**>gi555853:RibosomalDNA** **----.C...C............C..A.G......T.G...--.C............G......C..........G.......C.A....AGGCACAGCGC**

**fwd primer**  **----.C...C............C..A.G**

**rev primer**  **----------------------------------------------------------------------------------------------------**

**P.troglodytes**  **..................................................................................g.a....----------c**

110 120 130 140 150 160 170 180 190 200

....|....|....|....|....|....|....|....|....|....|....|....|....|....|....|....|....|....|....|....|

**H.sapiens**  **-TCGCTTTGGAAGCCACGGCACCGCCTTGTAAAGCCCCATTCATAAGCACAAAGCCCTATTCCCTTCCTGGAGTTGGAGCTGATGCCTTCCATAGCCTTG**

**AA166653**  **a...-..........g...a..n-.t..c-............g..t.....g....t..................................g........**

**>gi555853:RibosomalDNA** **A..............G.....A......TC............G..T.....G....T..................................G........**

**fwd primer**

**rev primer**  **----------------------------------------------------------------------------------------------------**

**P.troglodytes**  **a.....................a.............................................................................**

210 220 230 240 250 260 270 280 290 300

....|....|....|....|....|....|....|....|....|....|....|....|....|....|....|....|....|....|....|....|

**H.sapiens**  **GGCTTCTCTCCATTCAGAAGCTTTGACAGGGGCAACCCCACCCAGAGGCTGGCTGAGGCTGAGGATTACGGGGTGTGATGGGGCTGGAAAGTGGGTCCCC**

**AA166653**  **.n-............g..............c........................c..-.........g........t........a...c.........**

**>gi555853:RibosomalDNA** **...............G.......-......C...GGG..................C............G........T........A...C.........**

**fwd primer**

**rev primer**  **----------------------------------------------------------------------------------------------------**

**P.troglodytes**  **....................................................................................................**

310 320 330 340 350 360 370 380 390 400

....|....|....|....|....|....|....|....|....|....|....|....|....|....|....|....|....|....|....|....|

**H.sapiens**  **TATTTTTGATAGCTCAGCCAAGACATCCCCCGACCCCCATCGCTTGCTCACCCTTTGAGATCCCCCGCCTCCA-CCGCCTTGGAGGCTCACCTGTTACTT**

**AA166653**  **...........c.......g.c.............g.............g....c..................a........c..........c......**

**>gi555853:RibosomalDNA** **...........C.......G.C.............G.............G....C..................-........C..........C......**

**fwd primer**

**rev primer**  **----------------------------------------------------------------------------------------------------**

**P.troglodytes**  **.........................................................................-..........................**

410 420 430 440 450 460 470 480 490 500

....|....|....|....|....|....|....|....|....|....|....|....|....|....|....|....|....|....|....|....|

**H.sapiens**  **TAATTTCTGTCTTTCTCCCTTTCTTGCGTTTGAGGAGGGGGTGCAGGAATGAGGGTGTGTGTGGGAAGGGGGTGAGGGGTGGGGACGGAGGGGAGCGTCC**

**AA166653**  **.-......--------t................a..........g.........................-..tc.-..........a...--.a.....**

**>gi555853:RibosomalDNA** **.C......--------T...........................G....................G........C.........................**

**fwd primer**

**rev primer**  **------------------------------------------..G...............**

**P.troglodytes**  **....................................................................................................**

## Fig S5b. H. sapiens genome region: chr2:132864310-132864909


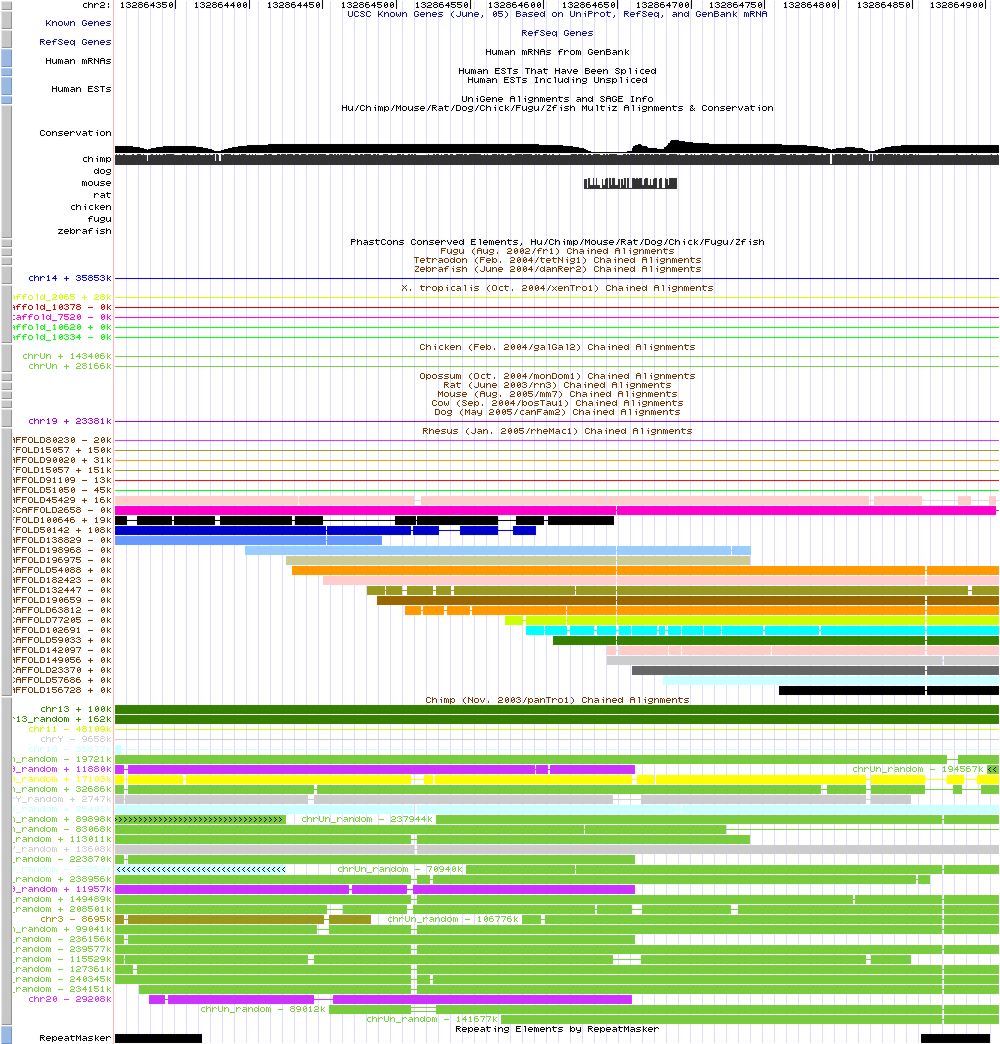


Fig S6a. HS.128594 H. sapiens genome region: chr3:54641157-54642780 + strand

10 20 30 40 50 60 70 80 90 100

....|....|....|....|....|....|....|....|....|....|....|....|....|....|....|....|....|....|....|....|

**H.sapiens**  **GAAAAGTTTTAGACAAATATGAAGAGAAAAGATATTACTCATCAAACCATTTGAATTTAATTGCATTCTTCAAAATTCTACAGCTACAG-ATTGCA-GCA**

**AI952931**  **----------------------------------------------------------------------------------------------------**

**fwd primer**  **---------------------------------------------------------........................**

**rev primer**  **----------------------------------------------------------------------------------------------------**

**P.troglodytes** **---------------------------------------------------------......................c.........-......-...**

**B.taurus**  **---------------------------------------------------------......................c.CAaCTgCAG.....tG-..**

**C.familiaris**  **---------------------------------------------------------..................-.TCT.C.aCTgCAG......GC-.**

**M.musculus**  **---------------------------------------------------------.......g...c......A..CT.tAagT.CAc.....-tt..**

110 120 130 140 150 160 170 180 190 200

....|....|....|....|....|....|....|....|....|....|....|....|....|....|....|....|....|....|....|....|

**H.sapiens**  **GAGAATA--CCAGGGACTCAGCCAGAGAAGAAAGTACTGTTTTTA--AATAAACAGCTCCTGGCAGCTCTTTATTCTAGTCCACATTATTCAGATATCTT**

**AI952931**  **--.....--....................................--.....................................................**

**fwd primer**

**rev primer**  **----------------------------------------------------------------------------------------------------**

**P.troglodytes** **.......--....................................--.....................................................**

**B.taurus**  **.....a.--...............tg..........a..ga....--..c.........gg......c.c..c...c...gtg..g..............**

**C.familiaris**  **.......--...................g......ga........--............ag......c.c..c...c....tgtg...............**

**M.musculus**  **...g...tg.a....g...t...t....g.....c..a..c...Gtt......tt........ggc...ga.T..TA.T-.....g.cc.......c.cc**

210 220 230 240 250 260 270 280 290 300

....|....|....|....|....|....|....|....|....|....|....|....|....|....|....|....|....|....|....|....|

**H.sapiens**  **CTTTTTTCCTCTCTTTCTTTCCATGTGTTTGGGAAAATTTTTCC-AGGATGTTTTCAGATGAGTTTGTG-AACAATGGCCCTAGAGTATTCTGTTTTTTT**

**AI952931**  **............................................-........................-..............................**

**fwd primer**

**rev primer**  **----------------------------------------------------------------------------------------------------**

**P.troglodytes** **............................................-............a...........-..............................**

**B.taurus**  **.A...c-............g..t.t....a....g.g.......-t..g........a.......aCaTG.....c..tttcgag..g.c...a......**

**C.familiaris**  **.A...c-............g..t.t.....c...g......g..-......c....cag.....CaCaTG.....ca.t.t..ag..g.ct..a......**

**M.musculus**  **a...C.CT.c.c----.......ct.tg.....c.tg...c.T.t.a...ag...tga..t....ccCAg..T.---....c..Tccc.g..C.c.----**

310 320 330 340 350 360 370 380 390 400

....|....|....|....|....|....|....|....|....|....|....|....|....|....|....|....|....|....|....|....|

**H.sapiens**  **CCCCTT--GAAGCTTCTGTGACCTGAAGCATAGATTACTTT-TATGCATTGGCTTGTTGCTGAGTTTATTCAATGGTGCGAAGCTGT-GTTTAATGCAGT**

**AI952931**  **......-ga.gct.ctgtgac.tga.gcatagat.act...a.gcattggcttg.tgctga.tt.a.tcaatggt.c.ang-tg.tgtt.aatgcagtc.**

**fwd primer**

**rev primer**  **-------------------------------------------------------------.TT.A.TCAATGGT.C.AAG**

**P.troglodytes** **-.....TGA.GCT.CTGTGAC.TGA.GCATAGAT.ACT...A.GCATTGGCTTG.TGCTGA.TT.A.TCAATGGT.C.AA**

**B.taurus**  **-.....cT.c...c......gt.aa.t.a.c....c....gTAT.Cgg.G.CacaT.aCTG.GT..ATcCAgTGacatGA.**

**C.familiaris**  **T...C.ttcTtcAGa..C..TGAaCTGAAGC...GAT.ACcT.TATtCAg..GGCccacTGCT.gGgT.ATTCAATGaTctGAA**

**M.musculus**  **-------GA.Gt..CTGTGcC.TGA.GCA-------------------ggcATgga.-----TT.A.tC.g.GGCT...TGt.Ggc.TTgca..gA.t.g**

## Fig S6b. H. sapiens genome region: chr3:54641157-54642780


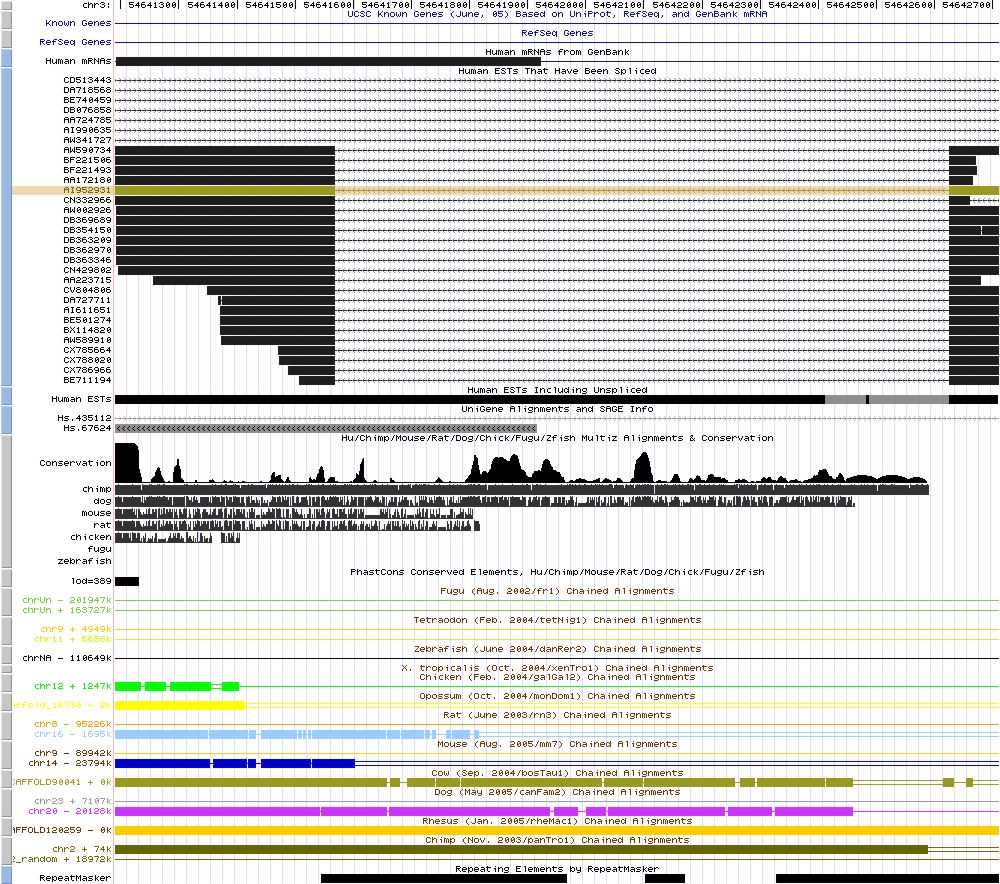

Supplement: Additional file 2 — Supplementary Information. Table S1. Cross-species alignment results for sequences within experimentally analyzed PCR fragments. Figures S3a, S4a, S5a, and S6a show BioEdit alignments of human genome sequences corresponding to fragments of interest with homologous sequences in the mouse and chimpanzee genomes. In Fig. S6a, homologs from cow and dog genomes are added. Primers that had been used for RT-PCR experiments are shown. The graphical alignments of the ESTs under study (set in the light yellow box in the pictures) with the human genome are present in Figures S3b, S4b, S5b, and S6b. Alignments of human genome fragments with corresponding genome fragments of other organisms (fugu, tetraodon, zebrafish, frog, chicken). [file 1750-9378-1-8-S2.doc]
